# Inpatient pain assessment and decision-making in internal medicine and general surgery residents: A qualitative analysis

**DOI:** 10.1016/j.heliyon.2024.e30537

**Published:** 2024-05-06

**Authors:** Robert C. Wright, Barbara J. Polivka, Jennifer A. Villwock

**Affiliations:** a Department of Otolaryngology, Head and Neck Surgery, University of Kansas Medical Center, 3901 Rainbow Blvd, Mailstop 3010, Kansas City, KS 66160, USA; b School of Nursing, University of Kansas Medical Center, 3901 Rainbow Blvd, Mailstop 2029, Kansas City, KS 66160, USA

**Keywords:** Pain assessment, Acute pain, Pain management, Resident, Resident physicians, Postoperative, Pain

## Abstract

**Background:**

Understanding physician approaches to pain treatment is a critical component of opioid and analgesic stewardship. Practice patterns learned in residency often persist longitudinally into practice.

**Objective:**

This study sought to identify salient factors and themes in how resident physicians assess and manage pain.

**Methods:**

Video-recorded focus groups of internal medicine and general surgery residents were conducted via videoconferencing software. Data were analyzed using a ground theory approach and constant comparative method to identify themes and subthemes. Focus groups occurred in September and October 2020.

**Results:**

10 focus groups including 35 subjects were conducted. Four general themes emerged: (1) Assessment considerations; (2) Education & Expectations; (3) Systems Factors; and (4) Management considerations. Participants indicated that while it is important to treat pain, its inherently subjective nature makes it difficult to objectively quantify it. The 0–10 numeric rating scale was problematic and infrequently utilized. Patient expectations of no pain following procedures was viewed as particularly challenging. The absence of formal best practices to guide pain assessment and management was noted in every group. Management approaches overall very highly variable, often relying on word-of-mouth relay of the preferences of specific attending physicians.

**Conclusions:**

Pain is highly nuanced and resident physicians struggle to balance pain's subjectivity with a desire to quantify and appropriately treat it. The 0–10 numeric rating pain scale, though ubiquitous, is problematic. Priority areas of improvement identified include education for both patients and physicians, functional pain scales, and expansion of existing effective resources like the nursing pain team.

## 1 Introduction

Understanding factors influencing inpatient pain assessment and management is critical to ensuring high quality patient care, including being good stewards of high-risk medications such as opioid-based analgesics. Opioids prescribed at hospital discharge often exceed patients' needs and are prescribed at the highest rates for high risk populations [1,2]. It was hoped that prescribing guidelines would decrease this variability while decreasing overall reliance on opioids. Unfortunately, existing guidelines are highly variable and focus on procedures performed rather than patients’ experiences [3].

The perspectives of resident physicians are particularly important since residency is a time when practice patterns are learned and solidified [4]. Educational interventions during this critical time could enhance patient care and modify problematic behaviors of inappropriate pain management, including both over- and under-prescribing [[5], [6], [7]]. Unfortunately, there is a gap in the literature regarding physician decision-making in this context. The existing literature is largely limited to expert opinion, survey data, or physician self-report of how they would respond to hypothetical situations [8]. Furthermore, studies of hypotheticals frequently omit a key component of pain – the patient experience. For example, a 2018 study of how residents would treat pain in four cases provided information only on patient age, surgery, and surgical indication [9]. Additionally, prior studies have revealed differences in the prescribing patterns of residents dependent on if they are training in surgery or medical specialties [10,11]. In the one qualitative study available of attending and resident surgeon approaches to postoperative pain prescribing, only perspectives about opioid prescribing – and not overall pain assessment and management – were described [12]. Data regarding how and why, and not just what, decisions are made by resident physicians is needed to inform educational initiatives to optimize pain management.

To address this gap, this study qualitatively analyzed focus group data of internal medicine and general surgery residents. The goal was to identify salient themes regarding how residents approach inpatient pain assessment and how management decisions are subsequently made. A secondary goal was to report differences in these approaches between surgical and internal medicine residents.

## 2 Methods

Qualitative Research Tradition: This research utilized the principles of grounded theory and was influenced by Strauss and Corbin's approach [[13], [14], [15]]. As such, no pre-existing code structure or existing theoretical framework was used to guide inquiry. However, authors were familiar with multiple theoretical frameworks regarding planned behavior and decision-making, which may have influenced how data was conceptualized and analyzed. JV was a clinically active otolaryngologist and BJP was the Associate Dean for the School of Nursing during the time this study took place. JAV and BJP are both women. Both have led prior qualitative research studies and completed graduate level coursework in qualitative methods. Interview transcripts were reviewed line-by-line and as concepts became apparent, they were assigned a code. Codes were compared and refined in an iterative process to develop the codebook. The codebook was revised to remove redundant codes, clarify concepts, and resolve disagreements between authors. Once the final codebook was agreed upon by both authors, the transcripts were again independently reviewed and fully coded. Data were further analyzed using the constant comparison method to ensure that newly assigned codes were consistent across focus groups and between reviewers. Credibility was established through data collection techniques and triangulation, ensuring the accuracy of our findings. We addressed transferability by providing detailed contextual information, allowing readers to determine the applicability of our results to other contexts. Confirmability was achieved by maintaining a systematic and transparent approach to data collection, analysis, and interpretation, ensuring that our findings are based on the participants' perspectives and experiences rather than researcher biases. Collectively, these measures ensure the dependability of our study, illustrating that our findings are consistent and replicable.

Setting: Resident physicians from the internal medicine and general surgery residency programs at a tertiary care academic hospital were invited to participate in focus groups. 30-minute focus groups occurred in September and October 2020. The lead author (JAV) sent a recruitment email to program residents informing them of the opportunity to participate in the qualitative study. Neither JAV nor BJP are involved in the provision of clinical or didactic training within the general surgery and internal medicine resident programs. The intent of the research – to better understand pain assessment and management from the trainees perspective – was presented to potential subjects prior to their enrollment. Purposive sampling was chosen in order to select resident physicians based on the research goal to better understand trainees' pain assessment and management perspectives. Interview questions were developed in an iterative fashion and underwent multiple rounds of review prior to finalization. Due to the limited size of potential participant pool – each academic medical center has a fixed number of resident physicians – pilot testing of interview questions was not feasible. Trained researchers who were not part of the subjects’ clinical training program conducted the focus groups. Due to the SARS-CoV-2 novel coronavirus (COVID-19) pandemic, all focus groups were conducted and recorded via videoconferencing software (Zoom, San Jose, CA) with participants utilizing appropriate private, quiet spaces. Focus groups were conducted during protected academic time with multiple focus groups occurring simultaneously and conducted by trained facilitators. The voluntary nature of the focus groups was emphasized and no record of attendance, other than what was institutionally required in order to provide subjects with $15 incentive, was kept. A semi-structured interview guide regarding approaches to inpatient pain assessment and management (Box 1) ensured consistency across groups. Contributions from subjects participating via chat function were read aloud by the facilitator. Because focus groups were conducted simultaneously, we were unable to conduct interim assessments of content and thematic saturation. Repeat interviews to further clarify emergent themes were not undertaken due to the time-intensive nature – 80-h work weeks – that the training study participants were undergoing. It was felt that follow-up interviews would be overly burdensome and may introduce bias regarding subjects who elected to participate.

Box 1 **Focus Group Discussion Guide**

● How do you decide how to manage a patient's pain? (Probes: pain assessments, discussions with patients, insight from other care team members)
● How do you determine if a patient's pain is appropriate or needs improved management? (Probes: discussion with patient or care team, what if patient disagrees with plan, patient expectations)
● How do you incorporate guidelines or best practices about pain management into practice? (Probes: what best practices exist, resources for complex patients, information given to patients, treatment options)
● What tools or resources would be helpful in assessing and managing pain? (Probes: different pain scales, education, shared decision-making aids)

Alt-text: Box 1

Data Analysis: Focus group recordings were autotranscribed via Trint (London, UK) artificial intelligence software. Accuracy of recordings was verified and transcripts uploaded into Dedoose (Hermosa Beach, CA). Each subject's contributions were individually coded by JAV and BJP and linked to descriptors (e.g., gender, self-identification of training level [e.g. “I'm an intern”]). Content and thematic saturation was reached and additional focus groups were deemed unnecessary.

The study protocol underwent institutional review board review and was approved (STUDY00142379) prior to commencement of any study materials. The COnsolidated criteria for REporting Qualitative research (COREQ) Checklist is available in Appendix 2.

## 3 Results

Thirty-five subjects (surgery = 20, internal medicine = 15) participated in 10 focus groups. This represents 80 % of the total general surgery and 22 % of the internal medicine residency cohorts. Focus group size ranged from three to six participants. No focus group participants dropped out once the session had begun. 44 % of surgical, and 40 % of internal medicine participants, identified as women. While the didactic sessions used to recruit participants were specific for residents, one participant in the internal medicine cohort – and two in the surgery cohort – self-identified as attending physicians during the focus groups. More specific age and race/ethnicity data are not presented to preserve participant anonymity. Due to technical issues, one general surgery focus group was unable to be recorded. This group was facilitated by the senior author, who has extensive qualitative experience. Researchers took field notes of their impressions and observations during and immediately following focus groups. As focus groups were all conducted synchronously during scheduled didactic time, researchers also met within 30 min of the conclusion of the focus groups to reflect on their experiences. These data were included in analyses.

Four general themes emerged and are listed in order of frequency of code application: (1) Assessment considerations; (2) Education & Expectations; (3) Systems Factors; and (4) Management considerations. Specific sub-themes were also identified (Table 1).

**Table 1:** Themes, subthemes and illustrative quotes.

Theme	Subtheme	Additional Illustrative Quotes
Assessment Considerations	Assessment strategies	“Usually, I just look at the patient as a whole. Look at the vital signs and just how the patient reacts on the physical exam. But yeah I usually rarely use, and actually, like, reliably use, the zero to 10 pain scale.” “One of the biggest things is obviously going to see the patient. Kind of just, without even talking to them, it's just kind of looking at their facial expressions, their body language, what they're doing when they're in bed. And then obviously examining their abdomen and seeing if they, you know, truly are tender to your touch or if it's just like the thought of us touching them, or kind of all of that is kind of what I look at, more cues than anything.”
	Subjectivity of Pain	“It's completely subjective, and it also just depends on whoever is assessing them, too. Because, to me, I may think that they're in much more pain than maybe [other participant] would or whatever. And so a lot of the way we do things is kind of at the discretion of whoever's doing the assessment. “
	Pain legitimacy	“A lot of patients are going to say they have 10 out of 10 pain. When you look at them, and, like, there's no way in heck you're having 10 out of 10 pain. You're laying here comfortably in bed. I would say at best you're probably six out of ten.” “Somebody could tell you, ‘my pain is at a 10’ and they're eating a cheeseburger. You know, that's different from somebody who has their pain at a 10 and they're writhing in bed.”
	Pain scales	“I have very strong feelings about this. I just think it's crazy that, you know, pain, it's like a fifth vital sign, because the whole point of vital signs is that their objective and pain is such a subjective measure. I don't have, like, a good solution for it, but I just don't see how, like, the one to 10 scale really like works because everyone's perception of pain is so different. Because we have patients who routinely get the same exact operations with very different pain requirements, you know, narcotics or non-narcotic use are primary in the hospital. And these are patients who are narcotic naive. I'm not including people who maybe may have addiction. These are patients who have never taken narcotics. It's just people's perception is different. So just the way we do things and the way we have everything set up around that is not helping patients or us.” “[W]e've probably all seen the patient where you go in the room and they report 10 out of 10 pain. And then they're actually, you know, laying on their bed, texting and laughing and watching videos. So kind of a little bit of assessment of the patient with their reported pain and kind of what you're observing. You know, are they grimacing or are they restless? Are they tachycardic? There's a few other things we look at just besides the patient reported pain score.” “Patients are tired of rating their pain on the 1–10 scale and they don't find it meaningful.”
Education and Expectations	Expectations	“I think a big part of pain control is also just kind of managing expectations. And so making sure that the patients know that they're not going to have zero pain after surgery, but that their pain should be at least manageable.” “… a lot of times I've heard from patients, you know, “I didn't think I would have pain post-operatively.” Even if it's pain that's different than what brought them into the hospital, a lot of times they just think that their pain is going to go away and they're going to magically feel better. So I think, setting expectations and then also just assessing pain level on patients postoperatively is very important.” “I think one thing that's super important to do is having, like, that expectation talk with our patients preoperatively. Because I feel like that definitely changes a lot of the course afterwards. If they know kind of what to expect and they understand that there's no way we're going to take away all their pain and that it's going to be something that they got to work with, then the type of pain they may have definitely kind of changes a lot of folks. People that weren't expecting that or didn't have that expectation … they sometimes do a lot worse.” “Some people are hesitant to speak out when they probably should because they're in more pain and they don't want to bother people. And other people are at a one or two by a more objective standpoint and they think it's, you know, poorly managed pain.”
	Education	“I know acupuncture is out there. I, I honestly don't know the studies or the research behind it, if it's beneficial or not. But I've heard from some patients and firsthand experiences that they've had good results with it or massages, physical therapy, obviously, and all sorts of other non-medication related modalities to treat pain as well.” “I don't know what is provided from the nursing perspective, but I always try and give my like, what I call the 3-min spiel on the way that pain medications work and the way that different pain medications work.”
	Discharge Preparation and Readiness	“Some patients tolerate pain better than others. So you'll have plenty who are just on Tylenol whenever they're leaving. So they don't need quite the prescription that others do. I think just keeping track of each patient individually can be helpful when you want to prevent, kind of overprescribing-prescribing, which is an issue that we have now.” “I usually, if they're on opioids, if I'm sending them home or discharging with opioids, I always give them the opioid education. I don't know how it comes, or in what form it comes to them in, but on discharge orders, I always check the opioid education information to send with the patient. I think it's instructions and kind of what they can expect when taking that class of medications.” “When you prescribe certain things for diabetics, that it'll prompt you if you want to have a diabetic educator see the patient. And there's nothing I've seen like that for opiates.”
Systems Factors	Formal protocols/best practices	“I've never had a staff present to me on a narcotic pain medicine regimen or some sort of pain regimen protocol.” “No protocols that I'm aware of, or at least that I can think of on the spot. I'm sure there are, but not that I have known about, or not that I am using at the moment.” “Use of acetaminophen is kind of the backbone of any pain regimen, if possible, especially in the inpatient setting … Starting with the baseline scheduled Tylenol and then having kind of an understanding of, OK, this is gonna be our foundation. And then for breakthrough pain, maybe we say, if your pain is greater than a seven or an eight out of 10, then we can, you know, we can use this opioid. And that way you're not kind of pigeonholing yourself into scheduling opioids, but you're saying, OK, we're going to limit this to only when the pain is above and beyond what is something that can be manageable to a patient so that they're also not getting scheduled opioid the entire hospitalization and then all of a sudden are expected to kind of wean themselves off at home. So that was one, I guess, that many attendings have kind of mentioned to me.” “I think that more of what our job is, at least coming off of nights, was kind of a bandage for the nights and not really following a protocol, but more so just word of mouth, what has worked in the past.”
	General Systems issues	“So, the discharge order set that we have includes a section for patient education. And probably the only one that I routinely click is the opioid educational button. I would be lying if I told you what, that I know what that actually includes. (laughs) But I'd certainly like to think that the health system has something reasonable to educate the patient on the meds they'll be taking at home if I click that.” “I usually, if they're on opioids, if I'm sending them home or discharging with opioids, I always give them the opioid education. I don't know how it comes, or in what form it comes to them in, but on discharge orders, I always check the opioid education information to send with the patient. I think it's instructions and kind of what they can expect when taking that class of medications. But past that, I don't know specifically of pamphlets or information we, as like a system here, give out to patients.” “I think the fact that we can do electronics scripts now, at least for me personally, has made me prescribe less narcotics because I don't have to worry about calling in and not being to have a script that truly need it. And because I know that one of us will always be available whether it's the clinic or one of the residents who's here to have them contact us when they're discharged.“ “I know that we generally recommend that patients follow-up with, like, their primary care provider, within a week of being in the hospital, but the numbers of that actually happening or following through, I feel like are probably not as high. And so I certainly think that's something that as a health care system in general, making those connections and trying to make sure there isn't a disconnect between this patient leaving the hospital with all their pain for this acute episode being controlled and that they're still dealing with chronic pain, having someone who is following them regularly or seeing them on a regular basis to make sure that that's resolving and getting better. That's something I think that continues to need improvement.”
	Role of nursing	“I think another problem is that I think, especially in the hospital … is like how nursing staff approaches pain with patients. I think it's like if you constantly are like, “oh, you know, you have these medications available … do you have pain, like how's your pain?” And I mean, it's not that it's their fault. It's just like how the system set up. That they're supposed to check the pain. And remind patients, “oh you have all these medicines available.” I think it just makes people think like, “oh, I need to take it.” Whereas if you're just, if you don't constantly bring it up, I don't know, at least, like, that's how I think a lot of people's psychology works. If you keep asking someone about something or bring it up, they start to think, “oh, it must be happening.” I think that could really contributes to the whole issue.”
Management Considerations	Service or procedure-specific approaches	“I don't know if I necessarily go off protocols to give them pain management. I more mainly go off, again, the type of procedure and the amount of dissection that we did. You know, if we put in like a port or something like that, which is just a little subcutaneous port for chemotherapy, it's not a big dissection. They don't necessarily need narcotic pain medications. “ “Yeah, a lot of what I've seen, you know, as you start off residency, is you figure out for certain types of operations, you know, there are certain pain regimens that are more typical to start on from upper level residents or staff.”
	Other resources	“There's one time I put in a consult for acute anesthesia pain and I called them and they said that you didn't need a really put a consult in. You can just call us before and discuss it through. And they gave me all these recommendations on how to really move to transfer a patient from, like, a PCA to oral pain med. So they're a good resource, but I only used it once.” “I mean, as an intern, I think it's hard to still understand, you know, I really rely on a lot of my upper levels to help me when I'm trying to decide, you know, someone actually needs more pain medication or if they just need maybe a different type of adjunct to help maybe target different receptors.”
	Difficult situations	“I would say the only thing that I feel like we tend to do is if someone has, like, an opioid history … I feel like we have a lower threshold to get the acute pain service on. And I just feel more comfortable with that because I think those patients require a little bit more finessing of exactly how to manage their pain. And I don't feels always super comfortable throwing on a lot of pain medication in that situation.” “Issues arise if the patient is not pain med naïve - then it's more challenging to manage.”

### 3.1 Assessment considerations

Assessing pain was universally noted to be problematic because it is an inherently subjective phenomenon. Participants noted the need to balance the type and level of pain they anticipated with the patient's stated experience of pain. The preferred method for pain assessment was to personally evaluate the patient, followed by vital sign review. Examples were given in every focus group where normal vital signs in the setting of verbal reports of extreme pain called into question the pain's legitimacy and need for treatment. Particularly among more junior residents, a common struggle was how to determine if a patient's pain was “real” without disrespecting the patient.

“You definitely have to be wise to how patients actually present with pain, because

you may have patients who have kind of narcotic addiction issues and who have, not necessarily malingering, but they're kind of very used to the health system. So, if they're like tachycardic and hypertensive, then, you know, they might be in pain. Whereas if they're saying your pain is 10, but they're sitting in bed with heart rate in the 60s and their blood pressure is normal and they're texting on their phone … then … yeah.”

Most participants were unaware of any formalized pain assessments other than the 0–10 numeric rating pain scale (NRS). Despite the ubiquity of the NRS, when describing it, several participants provided an incorrect lower value, calling it the “1–10” scale. The NRS was only felt to be helpful if the physician was already familiar with the patient and had insight into how that patient's pain was evolving over time. Several of the senior residents shared sentiments such as “I will say that I can personally count on one hand the number of times I've actually asked the patient to give me a numerical value for their score.”

The NRS was felt to introduce issues related to pain assessment and subsequent management considerations. On participant noted, “I think when you say to a patient “rate your pain on a scale,” the patient wants to make sure that you're aware that their pain is real. And, so, oftentimes they're saying these high numbers, but yet they're still able to eat, they're still able to walk, they're still able to do these functional day-to-day activities that would, in our mind say, hey, that's not a ten out of ten pain.”

### 3.2 Education/expectations

Education about pain control was felt to be lacking. For patients, this lack of education was felt to create issues around expectations for pain. It was mentioned in every focus group that a patient's expectation should not be zero pain. For surgical patients, robust pre-operative counseling was recommended to set pain and recovery expectations. It was also felt that patients with psychologic risk factors for heightened experiences with pain like poor coping strategies could benefit from pre-operative psychologic “pre-habilitation.”

In terms of medical education, little formal instruction on pain management was noted. When asked what would be helpful, participant responses included, “[a]ctually having classes in medical school about it [laughing from all participants]” and “I don't think we got enough education on it. It's, like, day one, you're on call. Of course, your first call is going to be like “hey, somebody's in this terrible pain.” And you're like, “well, do I use oxy? Like, what do I do?"

Desired ways to address these knowledge gaps and better align expectations included education for physician, nurses, and patients. Ideally, educational interventions to address these gaps would be actionable and guide subsequent steps in management.

“I think there's a lot of areas we have room for improvement … I think there needs to be some sort of move forward to try to educate patients on the pain scale and educate nursing staff and physicians, too, on how to appropriately use it and how it can be changed possibly. And making sure that we're looking at all the factors. Because there are many times when you're going to be called and you know, the patient could easily be asleep. And if you don't have the time, somebody else is crashing, you're just gonna keep adding pain medicines because you don't have time to go check on every patient.”

“I think developing honestly, if I had my way, I would do like an intensive, like, training with physicians and nurses to have them help develop a more complex pain scale. And I think the pain scale should use something like a picture or something like that so that we're adequately able to have patients who respond better to images and things like that.”

### 3.3 Systems issues

The most common systems issue was a lack of unified protocols or best practices regarding pain assessment and management. Several participants noted that they might exist, but they were not aware of them. Given lack of formalized protocols, most residents relied on procedure- or service-specific (e.g. breast surgery service/team) attending preferences relayed in a word-of-mouth fashion.

“It's like, ‘oh, well, we do it like this when we're on our surgical oncology rotation. But here we do it this way.’ So that constant changing of ways that we

prescribe things can definitely make it difficult, especially when it's early on and you're

kind of forming the way that you like to practice or the way that you like to manage

patients' pain.”

The surgical cohort recognized lack of post-discharge follow-up as a significant issue. While the importance of longitudinal follow-up was appreciated, residents were simultaneously aware that patients, especially those with underlying chronic pain, were probably not actually receiving this care. For example, “We generally recommend that patients follow-up with, like, their primary care provider, within a week of being in the hospital, but the numbers of that actually happening or following through, I feel like are probably not high.” In terms of feedback on their discharge prescribing, residents felt that they “almost never get feedback as to whether I gave too enough or too little [pain medication].”

The system-wide electronic health record (EHR) presented unique challenges and opportunities. Participants in most groups mentioned resources embedded in the EHR for patient education related to pain control such as templated language or “dotphrases” that can be easily incorporated into discharge instructions. However, no formal training was given on these resources. While many participants knew they existed, and some frequently used them, they did not know what information they contained.

“The discharge order set that we have includes a section for patient education. And probably the only one that I routinely click is the opioid educational button. I would be lying if I told you that I know what that actually includes. (laughs) But I'd certainly like to think that the health system has something reasonable to educate the patient on the meds they'll be taking at home if I click that.”

Nurses were viewed as a frontline source of accurate information about pain. One frequent frustration was nursing reliance on the NRS scale, particularly when the number was high, but pain was not adversely impacting the patient's function. There was also a feeling that nurses are forced to use this scale as a priority data point, despite widespread recognition of its subjectivity and associated problems. The linkage of common opioid orders with reported pain on the NRS was also felt to be problematic.

Existing health systems resources were noted to be helpful. The nursing pain team was mentioned as providing a valuable service, especially in the context of chronic or difficult pain. The only resident frustration was that the nursing pain team's focus was patient care and not resident education, further highlighting the need for formal pain education.

## 4 Management considerations

When asked about principles that guide their pain management, many participants – particularly in the surgical cohort – mentioned service- or procedure-specific approaches. However, this could also be a source of confusion. For example, many echoed the sentiment that “[s]ome people have the same exact procedure, can have much different pain scores, or pain evaluations, from that procedure. I think early on, I was definitely in the practice of, “oh, if you had a lap chole, you get this amount of pain medication."

When asked about specific situations in which pain was difficult to manage, many participants mentioned difficulty when reported pain and clinical context were not aligned. For example,

“I find it also interesting that there are some patients that come in and their scores are

consistently seven or eight no matter what you do. … It doesn’t seem to be a

coincidence that all of the narcotic pain medications, and the PRN breakthrough pain medications, are rated for seven or above.”

### 4.1 Differences between general surgery and internal medicine residents

the internal medicine focus groups discussed two topics that did not arise within the general surgery cohort. First, early in their management decision process, internal medicine resident emphasized the importance of reviewing current patient medications and other comorbidities. They emphasized the importance of being aware of any pre-existing liver or kidney pathology that this would influence appropriateness of medication selection were need to adjust the dose. The internal medicine cohort also repeatedly spoke about known frustrations in access to outpatient care. For example, particularly for patients with complex pain issues, while they would routinely write and discharge instructions that the patient should follow-up with an appropriate provider for continued pain management needs, they were aware that the majority of these patients would never receive these services. They expressed distress that this reality of healthcare and the downstream impacts may include readmission or other adverse outcomes.

## 5 Discussion

Understanding how resident physicians approach acute pain assessment and make decisions about treatment is critical to optimizing patient care and reducing inappropriate prescribing of high-risk medications like opioids. To address the gap in the literature regarding resident physician decision-making in this context, this qualitative study of general surgery and internal medicine resident focus groups identified salient themes impacting inpatient pain assessment and management. We identified four major themes – assessment considerations, education and expectations, system factors, and management considerations [12]. There was universal frustration with the NRS, including forced reliance on the NRS at a systems level to relay information and determine if medication could be administered. Determining the “legitimacy” or realness off a patient's pain, coupled with a lack of education in pain management, was a universal struggle for residents. Unrealistic patient expectations were identified as a major problem. Residents were in agreement that standardized education for physicians, nurses, and patients is needed to align expectations and allow for more meaningful communication regarding pain.

Among assessment considerations, legitimacy of the patient's subjective pain experience was the most frequently mentioned subtheme. Residents recognized that patients do have pain, but lacked effective methods for determining when pain should be treated. The NRS was viewed as only helpful if the resident was already familiar with the patient and, even then, often minimally so. The ambiguity inherent in treating pain was amplified when patients reported higher NRS values than expected. Residents struggled to balance two seemingly competing priorities – a desire to believe their patients and the belief that drug-seeking behavior often occurs.

The most common strategy to circumvent the subjective nature of pain was to consider the patient's vital signs as the primary objective indicator of pain. Participants in every focus group stated that they preferred to look at the patient's vital signs – not NRS data – to determine the significance of reported pain. This is potentially problematic as the validity of physiologic indicators of pain, like vital signs, are not adequately specific for pain detection [16]. While vital signs may change during the performance of a painful stimulus, such as turning a patient in bed, in conscious patients these changes were not associated with self-reported pain levels [17]. As a result, it is recommended that vital signs only be used as an indicator of pain when behavioral or verbal cues are not available, such as when a patient is mechanically ventilated or unconscious [17,18]. The residents in our study did not have consistent or effective methods for negotiating situations where patient reports of pain differed from what they expected. Resident did note to be “street smart” and “wise” to drug-seeking behavior, but no actual methods for determining next steps in management were offered.

Two aspects of pain management were conspicuously absent: nonpharmacologic options and shared-decision making. Responses to all questions about pain management focused on pharmacologic options, most commonly oxycodone, acetaminophen, or ibuprofen. Nonpharmacologic methods such as relaxation techniques, music, and distracting activities, these were never spontaneously discussed [19,20]. This is significant as prior studies of patients with acute pain in the emergency department setting have demonstrated that nearly half of all patients in pain did not desire analgesics [21]. Engaging patients in shared decision-making regarding pain was also not discussed. This is an important missing component as patient engagement has been shown to increase satisfaction with care and adherence to treatment recommendations [22,23]. Additionally, prior studies have shown that patients benefit both physically and psychologically from tools that help them self-manage their pain, particularly when they promote autonomy and self-empowerment [24,25]. Reliance on pharmacologic treatment of reported pain, plus lack education about nonpharmacologic options, may explain the lack of any references to physicians engaging in shared decision-making with patients in any of our focus groups.

In our study, participants indicated that expectations of no pain were pervasive among patients and very problematic. This is consistent with a 2020 study conducted by Schutte et al. demonstrating that 41 % of all patients, and 36 % of those undergoing major surgery, stated that “no pain” was a satisfactory pain level [26]. Surgical residents felt that more time pre-operatively should be dedicated to education regarding realistic expectations of post-operative pain. However, they were unsure with whom this responsibility rests, particularly given already limited clinician bandwidth. Indeed, while expectation setting with patients is recognized as a powerful method for influencing their subsequent postoperative experience [27] and potentially reducing issues with compliance and inadequate pain control [28], there is no clear “best way” to do so. Some residents suggested programs for pre-surgical physical and psychological optimization, similar to the cardiac prehabilitation patients often complete prior to complex cardiac surgeries. However, it was felt that it would not be reasonable for the surgeon to lead these efforts given other higher priority competing demands on their clinical time.

Education about pain management for physicians was universally felt to be lacking. Pharmacology taught in medical school often focuses exclusively on mechanisms of action and contraindications rather than how to incorporate these principles into clinical decision-making, potentially resulting in “inadequate and irrational prescription of drugs” [29,30]. Additionally, most pharmacology is taught in the preclinical years of medical school and prescribing competencies to be attained prior to graduation have yet to be developed [31]. Morone et al. note that pain education for physicians usually involves “piecemeal incorporation of pain topics into existing curricula or clinical rotations … The net effect has been a serious deficit in clinical skills for the evaluation and management of the patient in pain” [32]. Even when provided, training is not viewed as effective [33]. This is consistent with our finding that participants were unable to describe receiving any formal pain management training, even though they are expected to treat patients complaining of pain as first-year residents. Most relied on word-of-mouth relay of information from senior residents or attendings and rarely, if ever, receive actionable feedback on their prescribing or management patterns. This is consistent with prior studies showing that the most pervasive influence on prescribing behavior is attending preference. [7,34] Interestingly, within these studies, most residents report no direct opioid-related communication with their attending surgeons. For example, Gapsar et al. found that while 62 % of attending faculty report specifying prescription preferences to residents, only 9 % of residents noted this to routinely occur [34].

With respect to the health care system, residents unanimously and enthusiastically reported that the nursing pain service was helpful in the management of complex patients. However, it was also noted that because the focus of the service is the patient, not education, this was felt to be a missed opportunity to engage residents in pain management best practices. Residents in our study were aware of patient education materials related to pain within the electronic health record, none had personally reviewed this material or were aware of its content. This was true even for residents who frequently included such materials in their patients’ discharge instructions.

Given these findings, areas for improvement include enhanced education for both patients and clinicians. For example, many graduate medical education programs are incorporating quality improvement initiatives as a core requirement and could consider having residents rotate with already established nursing pain management and/or anesthesia teams to facilitate dissemination and implementation of interdisciplinary best practices. Given the reported reliance in our study on using vital signs to help objectify pain, resident education regarding the validity of this approach is also needed. Increasing familiarity with other pain assessments, particularly those that help objectify pain's impact on function, may be beneficial. While not yet widely implemented, functional post-operative pain assessments were found to better reflect patient pain, with specific functions – and not the NRS – being predictive of outpatient analgesic requirements [35]. Increased awareness of resources, such as the study sites nursing pain management team, would also be beneficial. Additionally, prior research shows that clinicians desire guidelines and simple interventions such as an opioid recommendation card for trainees is viewed as useful and does meaningfully change prescribing patterns [36]. However, as the aptly named article "It's Like Learning by the Seat of Your Pants”: Surgeons Lack Formal Training in Opioid Prescribing” notes, there are no widely applicable guidelines and most of these focus on procedures and do not incorporate the patient experience [37].

Our findings highlight several opportunities for future study. Many subjects referred to pain as a vital sign. This nomenclature has fallen out of favor, with entities like JHACO updating prior recommendations to focus on the functional impact of pain rather than treating a patient's reported numeric pain value. Given that our study population was physicians in residency training, research investigating methods of effectively disseminating and implementing more contemporary pain assessment priorities are warranted. Our participants also expressed frustration with the inherently subjective nature of pain and difficulties with assessing such a subjective phenomenon. As a result, and due to a desire to link pain to something objective, many used vital signs as an adjunct to determine pain's legitimacy. This approach can be problematic as vital signs have a wide range of what is considered normal; changes from baseline are more important than single measurements. Development, validation and deployment of more objective assessments of pain, such as the functional impact of pain, may help alleviate the problematic subjectivity of current methods.

## 6 Limitations

This study is not without limitations. All participants were residents from a single large academic medical center. As such, external validity may be limited. However, the overall resident demographic is similar to other programs of similar size throughout the U.S. and the academic setting includes numerous teaching faculty who trained outside of the study institution. There was some variability in focus group size, ranging from three to six participants. However, all focus groups lasted the same amount of time and similar themes emerged, so we do not consider this to be a significant limitation. Because focus groups were capped at 30 min to facilitate scheduling during ongoing protected academic time for residents, it is possible that additional themes would have emerged with more discussion time. Study participants did not review transcripts after the focus groups to verify accuracy; review of this nature was not feasible due to the number of participants and the time constraints of their residency training. Our sample size represents a convenience sample of surgical and medical residents who were present and willing to participate during their didactic time. Residents who were post-call, off-site, or who did not feel comfortable participating may have had unique perspectives, which would not have been captured. Additionally, despite reviewing inclusion criteria, three attending physicians remained in the focus groups. Their comments were not used as illustrative examples. In comparing transcripts between focus groups that did and did not include attending physicians, there were no differences in the number or frequency of applied codes and we do not believe that their participation significantly altered the discussions in those groups. While the principles of grounded theory were used in data collection and analysis, a novel theoretical framework was not developed. The primary aim of this study was to provide a detailed description and understanding of the phenomena under investigation, with a comprehensive depiction of the participants’ experiences and the contextual factors at play. While we recognize that advancing to a theoretical level of analysis could potentially provide deeper insights, the current study was designed with a focus on generating a rich descriptive understanding.

## 7 Conclusion

Assessment and management of pain is recognized as being both critical and highly complex and nuanced by both general surgery and internal medicine trainees. Trainees struggle with the concept of pain as a “vital sign” due to its inherent subjectivity and multiple factors that confound how and why pain is reported. Reliance on vital signs to better objectify pain is a common practice that is potentially problematic. Priority areas for improvement include improved pain education. For physicians, this should include instruction on pain-related clinical decision making. For patients, education about pain-level expectations and that “no pain” is not the goal should be priorities. Leveraging and expanding existing high value services like the nursing pain team to address these gaps as part of a collaborative, interdisciplinary solution is one potential approach.

## Ethical approval

This study was approved by the Institutional Review Board prior to commencement of any study activities.

## Informed consent

All subjects completed written informed consent.

## Ethics committee and approval number

University of Kansas Medical Center; STUDY00142379: ABC's of Pain.

## Funding

JAV is supported by a KL2 Career Development Award via the 10.13039/100007859University of Kansas Frontiers: Clinical and Translational Science Institute 10.13039/100016220CTSA Grant (UL1TR002366).

## Data availability

The codebook can be made available upon request.

## CRediT authorship contribution statement

**Robert C. Wright:** Writing – review & editing, Writing – original draft, Validation, Resources, Methodology, Investigation, Formal analysis. **Barbara J. Polivka:** Writing – review & editing, Writing – original draft, Software, Resources, Methodology, Investigation, Formal analysis, Data curation, Conceptualization. **Jennifer A. Villwock:** Writing – review & editing, Writing – original draft, Visualization, Validation, Supervision, Software, Resources, Project administration, Methodology, Investigation, Funding acquisition, Formal analysis, Data curation, Conceptualization.

## Declaration of competing interest

The authors declare that they have no known competing financial interests or personal relationships that could have appeared to influence the work reported in this paper.

## Figures and Tables

**Table:** 

No	Item	Guide questions/description	Response
Domain 1: Research team and reflexivity			
Personal Characteristics			
1.	Interviewer/facilitator	Which author/s conducted the interview or focus group?	Done It is mentioned in the methods
2.	Credentials	What were the researcher's credentials? E.g. PhD, MD	Done It is mentioned in the authors information
3.	Occupation	What was their occupation at the time of the study?	Done It is mentioned in the authors information
4.	Gender	Was the researcher male or female?	It is mentioned in the method
5.	Experience and training	What experience or training did the researcher have?	It can be inferred from authors information and also mentioned in methods
Relationship with participants			
6.	Relationship established	Was a relationship established prior to study commencement?	No
7.	Participant knowledge of the interviewer	What did the participants know about the researcher? e.g. personal goals, reasons for doing the research	At the beginning of each focus group, we explained the goals and reasons for doing the research. It is mentioned in the methods
8.	Interviewer characteristics	What characteristics were reported about the interviewer/facilitator? e.g. Bias, assumptions, reasons and interests in the research topic	Interviewers didn't report their own assumptions for not giving direction to the interviewees. But, we explained the personal goals, reasons for doing the research.
**Domain 2: study design**			
Theoretical framework			
9.	Methodological orientation and Theory	What methodological orientation was stated to underpin the study? e.g. grounded theory, discourse analysis, ethnography, phenomenology, content analysis	Principles of grounded theory It is mentioned in the methods section
Participant selection			
10.	Sampling	How were participants selected? e.g. purposive, convenience, consecutive, snowball	Purposive It is mentioned in the methods section
11.	Method of approach	How were participants approached? e.g. face-to-face, telephone, mail, email	Email It is mentioned in the methods section
12.	Sample size	How many participants were in the study?	35 It is mentioned in the results section
13.	Non-participation	How many people refused to participate or dropped out? Reasons?	0 It is mentioned in the results section
Setting			
14.	Setting of data collection	Where was the data collected? e.g. home, clinic, workplace	Zoom It is mentioned in the methods section
15.	Presence of non-participants	Was anyone else present besides the participants and researchers?	Attending physicians It is mentioned in the results section
16.	Description of sample	What are the important characteristics of the sample? e.g. demographic data, date	For confidentially reasons we just reported the organization, education and duration of professional activity of participants.
Data collection			
17.	Interview guide	Were questions, prompts, guides provided by the authors? Was it pilot tested?	Yes It is mentioned in the methods section
18.	Repeat interviews	Were repeat interviews carried out? If yes, how many?	No It is mentioned in the methods section
19.	Audio/visual recording	Did the research use audio or visual recording to collect the data?	Yes – Zoom It is mentioned in the methods section
20.	Field notes	Were field notes made during and/or after the interview or focus group?	Yes It is mentioned in the results section
21.	Duration	What was the duration of the interviews or focus group?	30 min It is mentioned in the method section
22.	Data saturation	Was data saturation discussed?	It is mentioned in the method section
23.	Transcripts returned	Were transcripts returned to participants for comment and/or correction?	No It is mentioned in the method section
**Domain 3: analysis and findings**			
Data analysis			
24.	Number of data coders	How many data coders coded the data?	There were two initial data coders in this study. All three authors were involved in finalizing codes
25.	Description of the coding tree	Did authors provide a description of the coding tree?	All codes consisted of Issues, Themes and Sub-themes which have been provided in Table 1.
26.	Derivation of themes	Were themes identified in advance or derived from the data?	Themes were derived from the data
27.	Software	What software, if applicable, was used to manage the data?	Trint and Dedoose It is mentioned in the methods section
28.	Participant checking	Did participants provide feedback on the findings?	No
**Reporting**			
29.	Quotations presented	Were participant quotations presented to illustrate the themes/findings? Was each quotation identified? e.g. participant number	Yes
30.	Data and findings consistent	Was there consistency between the data presented and the findings?	Yes
31.	Clarity of major themes	Were major themes clearly presented in the findings?	Yes
32.	Clarity of minor themes	Is there a description of diverse cases or discussion of minor themes?	Minor themes were highlighted in Table 1. Some discussion of minor themes is mentioned in the discussion.

## APPENDIX 2

“**Inpatient Pain Assessment and Decision-Making in Internal Medicine and General Surgery Residents: A Qualitative Analysis”**
